# A Comprehensive Microbial Gene Catalog of the Human Airway Microbiome Across Anatomical Sites and Geographic Regions

**DOI:** 10.1002/advs.76589

**Published:** 2026-07-27

**Authors:** Qing Zhang, Dashu Li, Bo Liu, Yue Zhang, Min Li, Ruochun Guo, Yawen Ni, Shibin Chen, Biao Ni, Lijie Qiu, Guorui Xing, He Dong, Qiulong Yan, Shenghui Li, Xiaohui Zou, Bin Cao

**Affiliations:** ^1^ Chinese Academy of Medical Sciences & Peking Union Medical College Beijing China; ^2^ National Center for Respiratory Medicine State Key Laboratory of Respiratory Health and Multimorbidity National Clinical Research Center for Respiratory Diseases Institute of Respiratory Medicine, Chinese Academy of Medical Sciences & Peking Union Medical College Department of Pulmonary and Critical Care Medicine Center of Respiratory Medicine China‐Japan Friendship Hospital Beijing China; ^3^ School of Basic Medical Sciences Tsinghua University Beijing China; ^4^ Department of Pulmonary and Critical Care Medicine Center of Respiratory Medicine Weifang People's Hospital Shandong Second Medical University Weifang Shandong China; ^5^ Puensum Genetech Institute Wuhan China; ^6^ Center For Microbiome Medicine The Fifth Affiliated Hospital of Southern Medical University Guangzhou China; ^7^ Changping Laboratory Chinese Academy of Medical Sciences & Peking Union Medical College Beijing China; ^8^ Department of Pulmonary and Critical Care Medicine Second Affiliated Hospital of Harbin Medical University Harbin China; ^9^ School of Mathematics and Statistics Changchun University of Technology Changchun China; ^10^ New Cornerstone Science Laboratory Department of Pulmonary and Critical Care Medicine Center of Respiratory Medicine China‐Japan Friendship Hospital Beijing China

**Keywords:** antibiotic resistance genes, antimicrobial peptides, gene catalog, human airway microbiota, virulence factor genes

## Abstract

The respiratory microbiota is a critical determinant of airway health, yet functional characterization remains challenging due to the lack of a high‐resolution reference catalog. To address this gap and enable systematic investigation at both species and gene levels, we constructed the integrated Human Airway Microbiome Gene Catalog (iHAMGC) through high‐throughput metagenomic analysis of 12,273 airway samples. This catalog comprises 24,185,985 non‐redundant microbial genes and provides extensive taxonomic and functional annotations, with a particular focus on clinically relevant elements, including antibiotic resistance genes, virulence factors, and antimicrobial peptides. We further resolved the bacterial hosts of resistance genes and virulence factor genes, as well as taxa contributing to antimicrobial peptide activity. The iHAMGC captures site‐specific microbial and functional variations across distinct airway niches and reveals regional differences in functional potential. By offering a comprehensive, publicly accessible reference for airway microbial genes, the iHAMGC serves as a foundational resource for advancing our understanding of the airway microbiota in respiratory health and disease.

## Graphical abstract

An integrated Human Airway Microbiome Gene Catalog (iHAMGC) is constructed from 12,273 metagenomic airway samples, providing a high‐resolution reference of respiratory microbial genes. The catalog enables systematic exploration of taxonomic and functional variation across airway niches and geographic regions, revealing site‐specific and region‐associated microbial functions, including antibiotic resistance, virulence factors, and antimicrobial peptides, with broad implications for respiratory research.

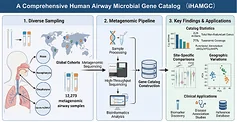

## 1 Introduction

The airway serves as the primary interface between the human body and the external environment, playing an essential role in gas exchange, physiological homeostasis, and defence against infectious agents [1, 2]. With the rapid advancement of microbiome research, the airway microbiota, comprising bacteria, fungi, and viruses, has garnered increasing attention for its contributions to respiratory health [3, 4]. These microbial communities participate in immune system maturation, modulation of inflammatory responses, and protection against pathogen colonization [5, 6]. Conversely, disruption of the airway microbial ecosystem, or dysbiosis, has been implicated in a range of respiratory conditions, including asthma, chronic obstructive pulmonary disease (COPD), cystic fibrosis, and severe respiratory infections [7, 8, 9].

Despite these advances, current knowledge of the airway microbiome remains constrained by several important limitations [10]. Many existing studies have focused on specific disease contexts, individual anatomical sites, or geographically restricted cohorts, making it difficult to distinguish robust microbial signatures from cohort‐specific or site‐specific artifacts [11]. Furthermore, the upper and lower airways differ substantially in microbial biomass, host DNA content, environmental exposure, and sampling accessibility [10, 12], yet many available resources fail to adequately capture this anatomical heterogeneity. Importantly, the majority of airway microbiome investigations have emphasized taxonomic composition, while gene‐level and functional diversity, including antibiotic resistance genes (ARGs), virulence factor genes (VFGs), antimicrobial peptides (AMPs), and other functional elements, remain insufficiently characterized across airway sites and populations. These gaps hinder the utilization of metagenomic reads, limit functional interpretation, and reduce cross‐cohort comparability in respiratory microbiome research.

To support gene‐level investigation of the respiratory microbiome, the integrated Respiratory Microbial Gene Catalog (RMGC) was previously established, comprising approximately 2.25 million non‐redundant microbial genes derived from 247 children [13]. While the RMGC provided a valuable reference for uncharacterized microbial components and host‐microbe interactions, its utility was inherently constrained by the narrow demographic scope and reliance on upper respiratory samples. This lack of anatomical and geographic representation limited its generalizability, particularly for studies involving lower airway niches or diverse global populations. Thus, there remains a critical need for a comprehensive, high‐resolution airway microbial gene catalog spanning multiple anatomical sites and geographic regions to fully capture the taxonomic and functional diversity of the human airway microbiome.

In this study, we presented the integrated Human Airway Microbiome Gene Catalog (iHAMGC), a large‐scale resource designed for in‐depth characterization of respiratory microbial composition and function. By integrating 12,273 metagenomic samples from 11 airway‐related sampling sites and 17 countries, we constructed a catalog comprising 24,185,985 non‐redundant genes. Compared with previous resources, the iHAMGC substantially expands the anatomical and geographic coverage of airway metagenomic data and provides a broader reference framework for taxonomic profiling, functional annotation, and cross‐cohort comparison. Using this catalog, we further characterized microbial heterogeneity across airway niches and populations, assessed functional features including ARGs, VFGs, and AMPs, and evaluated how sampling site, geographic origin, and available host background information contribute to airway microbiome variation. Collectively, the iHAMGC offers a scalable reference resource for respiratory microbiome studies and facilitates future investigations into microbial biomarkers, host‐microbe interactions, and respiratory disease.

## 2 Methods

### 2.1 Data Description

To construct the iHAMGC, we curated a collection of 12,273 global metagenomic samples, including 2,673 newly sequenced in house samples and 9,600 from public databases (Table S1). After rigorous quality control and removal of human host sequences, this resource yielded approximately 20.2 Tb of high‐quality, paired‐end sequencing data, with an average depth of 1.7 Gb per sample (Table S2). These samples covered 11 sampling sites, including nose, nasopharynx, oropharynx, throat, cough swabs, supraglottic aspirate, sputum, tracheal aspirate, bronchoalveolar lavage fluid (BALF), lung tissue, and other mixed samples (Figure 1A). They were obtained from 17 countries, with the majority from China, the United States (US), and Germany, among others.

**FIGURE 1:**
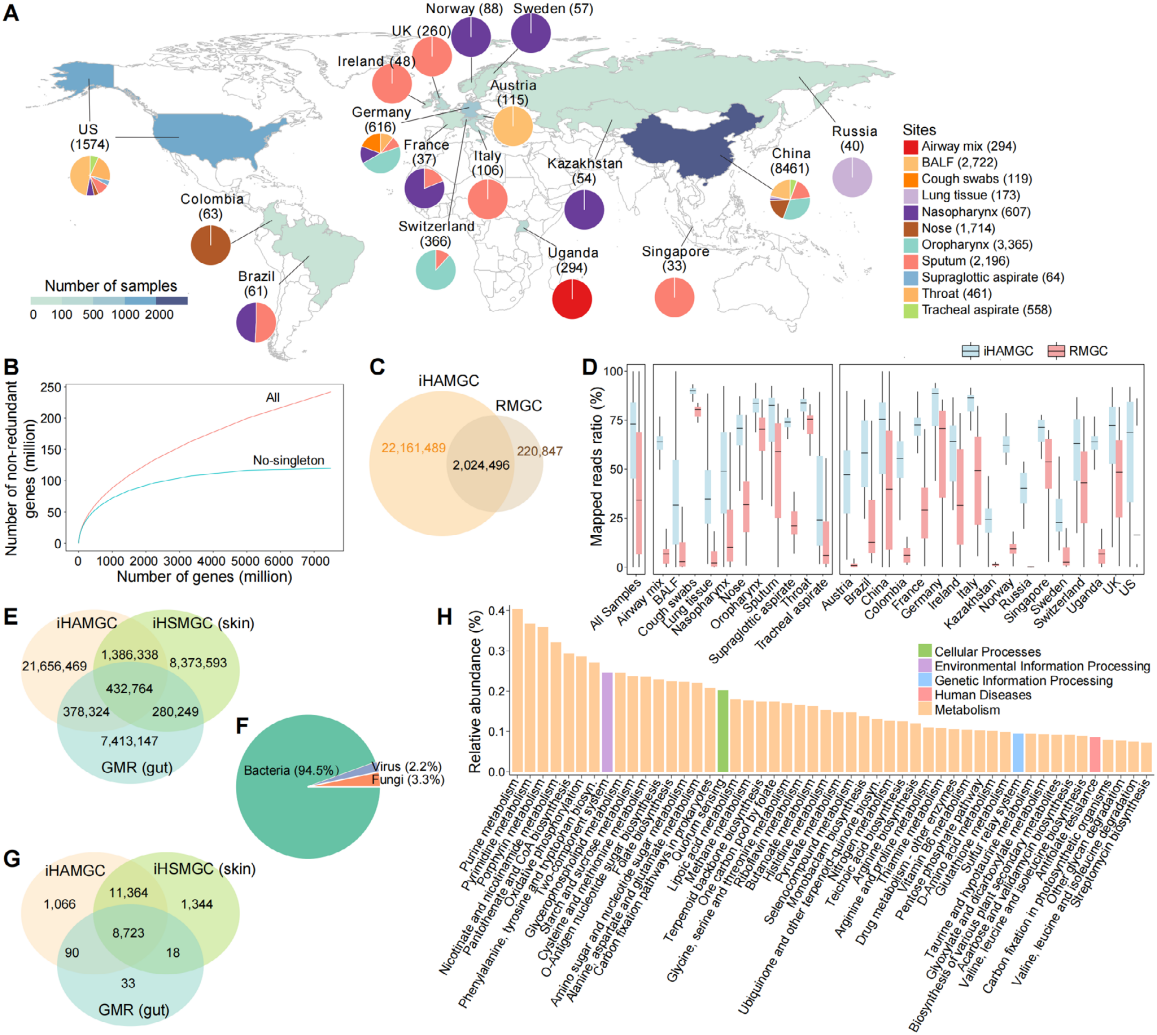
Construction and characterization of the respiratory gene catalog (iHAMGC). (A) Global sample distribution and sampling site information. (B) Saturation curve of non‐redundant genes during the construction of the gene catalog. (C) Venn diagram showing the gene overlap between iHAMGC and the existing respiratory gene set RMGC. (D) Comparison of mapped read ratios between iHAMGC and RMGC across different sample types. (E) Venn diagram illustrating overlapping and unique non‐redundant genes among the respiratory, skin, and gut microbial gene catalogs. Numbers indicate gene counts per region. (F) Pie chart showing the composition of biological domains (prokaryotes, eukaryotes, viruses) in iHAMGC. (G) Venn diagram showing the distribution of shared and unique species annotations among the three body sites. (H) Bar plot of relative abundances of KEGG pathway levels in the iHAMGC.

### 2.2 Gene Catalog Construction

To construct the airway microbiome gene catalog, sequencing reads from this study were processed through a comprehensive pipeline comprising quality control, host read removal, *de novo* assembly, and gene prediction. First, adapter and low‐quality or complexity sequences were removed using fastp (v0.23.4) and BBDuk (v39.0079) [14, 15]. Next, to eliminate host contamination, reads were aligned to the human reference genome (CHM13v2.0) using Bowtie2 (v2.4.4) [16, 17], and matched reads were discarded. The remaining high‐quality reads were assembled into contigs using MEGAHIT (v1.2.9) with a multi‐k‐mer strategy (kmer list: 21, 41, 61, 81, 101, 121, 141) [18]. Ab initio gene prediction was performed on all assembled contigs using Prodigal with the parameter “‐p meta” [19], resulting in the identification of 733,009,220 putative protein‐coding genes. The predicted genes were subsequently clustered at the nucleotide level using the easy‐cluster module of MMseqs2 (v12.113e3) with the following parameters: “–min‐seq‐id 0.95 –cov‐mode 1 ‐c 0.9 –cluster‐mode 2 –cluster‐reassign 1 –kmer‐per‐seq 200 –kmer‐per‐seq‐scale 0.8” [20]. Genes sharing more than 90% sequence overlap and 95% sequence identity were considered redundant. This process yielded a non‐redundant gene catalog comprising 24,185,985 reference genes.

To systematically compare the newly constructed iHAMGC (respiratory microbial gene catalog) with existing catalogs from other body sites, we integrated data from the Human Gut Microbiome Reference (GMR) [21] and the integrated Human Skin Microbial Gene Catalog (iHSMGC) [22]. For the gut dataset, genes were predicted from high‐quality genomes in the GMR using Prodigal, and non‐redundant gene sets were generated by clustering nucleotide sequences at 95% identity using MMseqs2. The skin microbial gene catalog was directly obtained from the iHSMGC dataset. Comparative analysis across the three body sites (respiratory, skin, and gut) was performed by clustering their respective gene catalogs using MMseqs2.

### 2.3 Taxonomic Classification and Functional Annotation of Genes

Taxonomic classification of non‐redundant genes catalog (24.2 million genes) was performed by searching against the NCBI‐NT database (downloaded in June 2025) using MMseqs2 with parameters ‘easy‐taxonomy –tax‐lineage 1 –lca‐mode 2 –max‐seqs 100 ‐e 0.00001 ‐s 6 –max‐accept 100’. A minimum alignment coverage of 70% per gene was required. The taxonomic assignments were made based on the best hit, with 70.73% (n = 17,106,093) genes successfully annotated.

For functional characterization, non‐redundant genes were annotated against the Kyoto Encyclopedia of Genes and Genomes (KEGG) database [23], the Virulence Factor Database (VFDB) [24], and the Comprehensive Antibiotic Resistance Database (CARD) [25]. KEGG Ortholog (KO) assignments were performed using DIAMOND (v2.0.13) [26] blastp with minimum query coverage of 50% and a minimum alignment score of 60. ARGs were identified by aligning protein sequences to the CARD using RGI (v6.0.3) with default parameters. VFGs were identified by alignments with the VFDB using DIAMOND, with criteria of >90% sequence identity and >80% query coverage. AMPs were predicted using the large language model ProteoGPT (version 3) [27]. Sequences with a prediction probability exceeding 90% were selected as putative AMPs.

### 2.4 Quantification of Genes

The abundance of each gene was quantified across all samples. Clean reads were aligned to the reference genes using Bowtie2 with default parameters [28]. Read counts were normalized to transcripts per kilobase million (TPM) to account for differences in sequencing depth and gene length.

### 2.5 Statistical Analysis

All statistical analyses were performed in R (v4.3.1). Alpha diversity was assessed by calculating observed richness and the Shannon diversity index from species‐level relative abundance profiles. Between‐group differences were evaluated using the Wilcoxon rank‐sum test. Beta diversity was assessed using Bray‐Curtis distances computed with the *vegan::vegdist *function (v2.6.8) [29]. Based on this distance matrix, permutational multivariate analysis (PERMANOVA) was performed using the *vegan::adonis2* function, and the obtained R^2^ values were further adjusted using the *RsquareAdj* function. Principal coordinate analysis (PCoA) was carried out using the *ape::pcoa* function, and the resulting plots were generated with *ggplot2* (v4.0.0). To identify differentially abundant taxa across anatomical sites and geographic regions, we first filtered microbial features with a relative abundance exceeding 0.05% in the respective groups. Multivariate association linear models (MaAsLin2) (v4.4.2) were then applied [30], adjusting for potential covariates including sex, age, and body mass index (BMI). The resulting phylogenetic tree and abundance heatmap were visualized using iTOL [31].

To evaluate pairwise relationships between AMPs and microbial species, Spearman correlation analysis was performed. P values were adjusted for multiple testing using the Benjamini Hochberg (BH) method via the p.adjust function in R. Significant negative correlations were defined as those with an absolute Spearman correlation coefficient greater than 0.40 and an adjusted q value below 0.05.

## 3 Results

### 3.1 Construction of iHAMGC and Cross‐body Site Comparison

To construct a comprehensive catalog of the human airway microbial genes, we collected 12,273 global metagenomic samples, including 9,600 samples retrieved from public databases and 2,673 newly sequenced in‑house samples (Tables S1 and S2). These samples represented 11 anatomical sites, spanning oropharynx (n = 3,365), BALF (n = 2,722), sputum (n = 2,196), nose (n = 1,714), nasopharynx (n = 607), and throat (n = 461), among other sites (Figure 1A). They originated from 17 countries, with the majority from China (n = 8,461), the US (n = 1,574), and Germany (n = 616), among others.

Following standard metagenomic processing, we obtained over 733 million genes from the airway metagenomic samples. These genes were subsequently clustered at 95% nucleotide identity into 24,185,985 non‐redundant genes, of which 49.5% (n = 11,981,391) were non‐singleton genes. The accumulation curve approached saturation as gene number increased, underscoring the representativeness of iHAMGC for the human airway microbiome (Figure 1B). Compared with the existing airway gene catalog (RMGC), iHAMGC covers 90.2% (2,024,496/2,245,343) of RMGC genes, while 91.6% (22,161,489/24,185,985) of iHAMGC genes are novel and absent from RMGC (Figure 1C). These results indicate that iHAMGC expands the airway gene repertoire by approximately 11 fold relative to RMGC. Notably, iHAMGC demonstrated substantially improved read‐mapping performance over RMGC across diverse anatomical sites and geographical regions (Figure 1D). The overall median mapping rate was 73.0% across all samples, markedly surpassing that of RMGC (34.3%). Stratified by anatomical site, the improvement was particularly pronounced in lung tissue (iHAMGC vs. RMGC: 34.8% vs. 2.1%), BALF (31.7% vs. 2.7%), nasopharynx (48.9% vs. 10.1%), and tracheal aspirate (24.0% vs. 5.9%). Across geographic regions, iHAMGC consistently yielded higher mapping rates than RMGC. The largest relative gain was observed in the US, where the mapping rate increased from 16.4% to 68.7%; in China, it rose from 39.8% to 75.6%; in Germany, from 70.8% to 88.5%; and in other countries, from 25.5% to 58.8%.

To investigate functional divergence among microbial communities across distinct human body habitats, we systematically compared the gene catalogs derived from the respiratory tract (iHAMGC), skin (iHSMGC) [22], and gut (GMR) [21] (Figure 1E). The comparison revealed strong site specificity in gene pools. The respiratory tract harbored the most unique genes (21,656,469; 90.8% of its catalog), far exceeding skin (8,373,593; 79.2%) and gut (7,413,147; 87.2%). Respiratory and skin shared 1,386,338 genes (5.8% and 13.1% of their respective pools), whereas a three site core of 432,764 genes accounted for only small fractions (1.8%, 4.1%, and 5.1% of each catalog). Taxonomic annotation against the NCBI‐NT database successfully classified 70.7% (17,106,093) of the non‐redundant genes, among which bacteria dominated (94.5%), followed by fungi (3.3%) and viruses (2.2%) (Figure 1F). In stark contrast to this marked genetic divergence, we observed considerable cross‐habitat commonality at the taxonomic level (Figure 1G). Specifically, a large core species pool was shared across the three datasets, with 8,723 species co‐occurring in the respiratory tract, skin, and gut, accounting for ∼41.1%, ∼40.5%, and ∼98.4% of their respective total species catalogs. Notably, the respiratory and skin microbiomes displayed exceptionally high species connectivity, sharing 11,364 species—far surpassing the number of site specific species (1,066 in the respiratory tract and 1,344 in the skin, representing ∼5.0% and ∼6.2% of their total species pools, respectively). Conversely, the gut microbiome had very few unique species (only 33), with the vast majority of its species shared with the other two sites. This pattern of “genetic heterogeneity but taxonomic homogeneity” suggests that, although microbial communities at different body sites may acquire site‐adaptive genes via horizontal gene transfer or strain‐level variation, extensive microbial exchange or shared niche preferences likely occur among human body sites at the level of species colonization.

### 3.2 Functional Annotation of iHAMGC and Microbial Functional Diversity

Functional annotation of the iHAMGC gene revealed distinct patterns of functional specificity and conservation across bacterial, fungal, and viral genes (Figure S1A). In terms of domain‐unique functions, bacteria contributed the largest repertoire (7,470 KOs, 69.8% of all unique KOs), followed by fungi (3,208 KOs, 30.0%), whereas viruses had only a minimal set (20 KOs, 0.2%). Regarding shared functions, the most extensive overlap occurred between bacteria and fungi, with 2,764 common KOs (19.6% of the total unique pool), indicating substantial metabolic convergence between these two kingdoms. A core set of 274 KOs was conserved across all three domains, accounting for 3.7%, 8.5%, and 91.3% of the bacterial, fungal, and viral unique repertoires, respectively. Notably, cross‐kingdom sharing involving viruses remained limited, with only 381 KOs shared between bacteria and viruses (3.6% of the total) and 19 between fungi and viruses (0.2%).

Further functional analysis against the KEGG catagories revealed the primary adaptive and metabolic potential of the airway microbial community (Figure 1H). Metabolism emerged as the most prominent functional category, within which purine, pyrimidine, and porphyrin metabolism pathways exhibited the highest relative abundances. Beyond core metabolism, other key modules were also enriched. For example, two‐component systems (involved in environmental information processing) indicate that these microbes can sense external signals and regulate nutrient uptake, while ribosome‐related genes (cellular processes) support basic protein synthesis. Finally, although present at relatively lower proportions, pathways linked to human diseases (e.g., antifolate resistance) and specific biosynthetic routes were also detected. This implies that, in addition to sustaining their own survival, the airway microbiome may influence host health through potential pathogenic mechanisms or metabolic interactions.

PERMANOVA based on KO profiles revealed that disease status significantly influenced overall microbial community functions across sampling sites (*p* < 0.001), although the magnitude of this effect varied considerably across countries. Specifically, the effect was substantial for samples from Switzerland, but weak or not significant for those from the US and Italy (Figure S1B,C). Further analysis of functional diversity showed significant differences in gene richness and evenness across countries and anatomical sites (*p* < 0.05 to *p* < 0.0001), highlighting strong geographic and local environmental influences on metabolic potential (Figure S1D). PCoA confirmed a clear separation of functional profiles by both country and sampling site (*p* = 0.001), with the highest explanatory power observed for US samples (R^2^ = 33.7%) and throat samples (R^2^ = 17.0%) (Figure S1E).

### 3.3 Species Composition, Diversity, and Differential Characteristics of the Respiratory Microbiota

Leveraging sufficient sample size and consistent coverage across datasets, we examined microbial composition and function at three shared airway sites (sputum, nasopharynx, throat) in three countries (China, US, and Germany), aiming to characterize species composition, diversity, and differential features across anatomical regions and geographic locations. We analyzed 2,356 airway samples collected from three distinct anatomical sites in three countries. To assess the effects of disease status and geographic location, we performed PERMANOVA at the species level and conducted comprehensive taxonomic profiling of bacteria, fungi, and viruses. Our results revealed that the effects of disease status were site‐specific, with the strongest impacts observed in oropharynx, sputum, and BALF, and geographically heterogeneous, particularly between Switzerland and the UK (Figure S2A,B). Taxonomic profiling revealed pronounced differences in microbial community structure across airway sites and countries. For the bacterial component, we first examined anatomical site effects. Nasopharynx samples exhibited a unique profile characterized by enrichment of potential opportunistic taxa, most notably *Dolosigranulum pigrum* (3.9%) and *Clostridium* *sp*. (3.6%) (Figure 2A). In contrast, throat communities were dominated by oral‐associated commensals and anaerobes, including *Prevotella histicola* (4.0%), *Prevotellaceae* *bacterium* (3.8%), and *Actinomyces graevenitzii* (3.6%). Sputum samples, representing the lower respiratory tract, displayed a more heterogeneous community with contributions from oral‐derived and mucosa‐associated taxa, including *Rothia mucilaginosa* (3.3%), *A. graevenitzii* (2.8%), and *P. histicola* (2.6%). Geographically, distinct compositional signatures were also observed among national cohorts (Figure 2B). The Chinese cohort more heterogeneous community with contributions from oral‐derived and mucosa‐associated taxa, including *R. mucilaginosa* (2.7%) and *A. graevenitzii* (2.4%). The US cohort was characterized by a predominance of oral‐associated taxa, including *P. histicola* (3.5%), *R. mucilaginosa* (3.1%), and *Prevotellaceae* *bacterium* (2.6%). The German cohort exhibited a diverse, mixed signature of oral‐derived and mucosa‐adapted taxa, with *P. histicola* (4.7%) and *A. graevenitzii* (4.6%) emerging as the predominant species.

**FIGURE 2:**
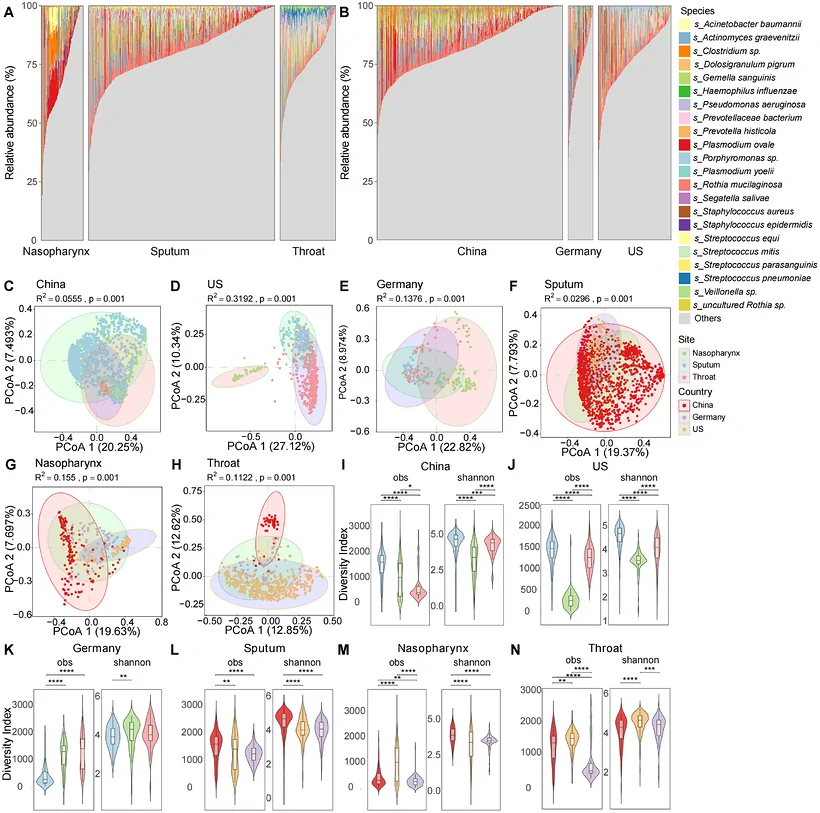
Species‐level composition and diversity analysis of the iHAMGC geneset. (A) Stacked bar plots of species composition across three respiratory sampling sites (Sputum, Nasopharynx, Throat). (B) Stacked bar plots of species composition across three countries (China, US, Germany). (C–E) PCoA plots based on Bray‐Curtis distances for different sampling sites within each of the three countries. (F–H) PCoA plots based on Bray‐Curtis distances for different countries within each of the three sampling sites. (I–K) Comparisons of species alpha diversity (Observed features and Shannon index) across different sampling sites within each country. (L–N) Comparisons of species alpha diversity across different countries within each sampling site. Asterisks indicate statistically significant differences (*p < 0.05, **p < 0.01, ***p < 0.001, ****p < 0.0001).

Similarly, fungal community composition was shaped by both anatomical niche and geography (Figure S2C,D). *Mucoraceae* dominated overall but varied significantly across sites: it peaked in the throat (55.6%) and sputum (39.2%), while the nasopharynx was distinctively enriched with skin associated *Malasseziaceae* (32.3%). Geographically, the US cohort showed the highest *Mucoraceae* abundance (43.0%), whereas Germany exhibited a more balanced profile with elevated *Malasseziaceae* (15.8%). China showed high levels of *Mucoraceae* (37.3%) and *Glomeraceae* (13.7%) but the lowest *Malasseziaceae* (5.2%).

For the viral community (virome), a substantial proportion of reads across all sites remained unclassified (“Others”) (Figure S2E,F). Despite this pervasive “dark matter”, specific viral families demonstrated clear site specific enrichment. The nasopharynx exhibited the most diverse detectable virome, dominated by *Orthoherpesviridae* (7.1%), *Polyomaviridae* (5.0%), and *Adenoviridae* (3.3%). In contrast, sputum and throat viromes were less diverse but highly enriched in bacteriophages, particularly *Herelleviridae* (2.8% in sputum; 3.0% in throat) and *Drexlerviridae* (2.3% in sputum). Geographical analysis further revealed regional variations. For instance, *Orthomyxoviridae*, often associated with influenza, was notably present in samples from China (3.9%) and Germany (3.6%), while *Herelleviridae* exhibited higher abundances in Germany (4.6%) and the US (3.0%).

PCoA confirmed significant spatial separation of microbial communities among sites within each country (Figure 2C‐E). The strongest separation was observed in the US (R^2^ = 31.9%), followed by Germany (R^2^ = 13.8%) and China (R^2^ = 5.6%). When stratified by anatomical site (Figure 2F‐H), the nasopharynx exhibited the highest geographic differentiation (R^2^ = 15.5%); in contrast, sputum showed lower but still significant geographic separation (R^2^ = 3.0%), suggesting that the lower airway microbiome is more conserved across populations.

For bacterial diversity, sputum samples consistently exhibited significantly higher richness and Shannon diversity than nasopharynx and throat samples across all countries, except in Germany (Figure 2I‐K). Across all sites, German cohorts generally displayed lower microbial richness compared to Chinese and US cohorts (Figure 2L‐N). For fungal communities, China showed the highest richness in sputum and throat samples. In contrast, Shannon diversity was generally lower in nasopharynx samples compared with other sites across China and the US (Figure S2G). Viral diversity varied significantly by anatomical site and country: sputum samples had the highest richness and Shannon diversity in China and the US, whereas nasopharynx samples displayed the highest diversity in Germany (Figure S2H).

We further identified taxon‐level signatures that characterize each sampling site. In total, 298 species were differentially abundant across airway sites (Figure 3A), and 297 species showed differential abundance across geographical regions (Figure 3B), indicating that microbial composition is highly site‐specific and geographically structured. Phylogenetic clustering and heatmap analysis revealed distinct biogeographical patterns. At the genus level, bacterial taxa exhibited significant spatial heterogeneity driven by both host anatomy and geographic location. Regarding anatomical site specificity, bacterial generaincluding *Streptococcus*, *Actinomyces*, *Prevotella*, and *Neisseria* consistently showed lower abundance in the nasopharynx and higher enrichment i n sputum or throat compartments across all three countries (Figure 3A). Conversely, most fungal species exhibited an inverse niche preference, being predominantly enriched in the nasopharynx rather than in lower airway or oropharyngeal sites. Geographically, *Streptococcus* and *Neisseria* were consistently more abundant in the US samples than in other countries, whereas *Actinomyces* showed a distinct preference for Chinese cohorts. Fungal and viral communities displayed an opposite pattern, with higher prevalence in China and Germany but marked depletion in the US (Figure 3B).

**FIGURE 3:**
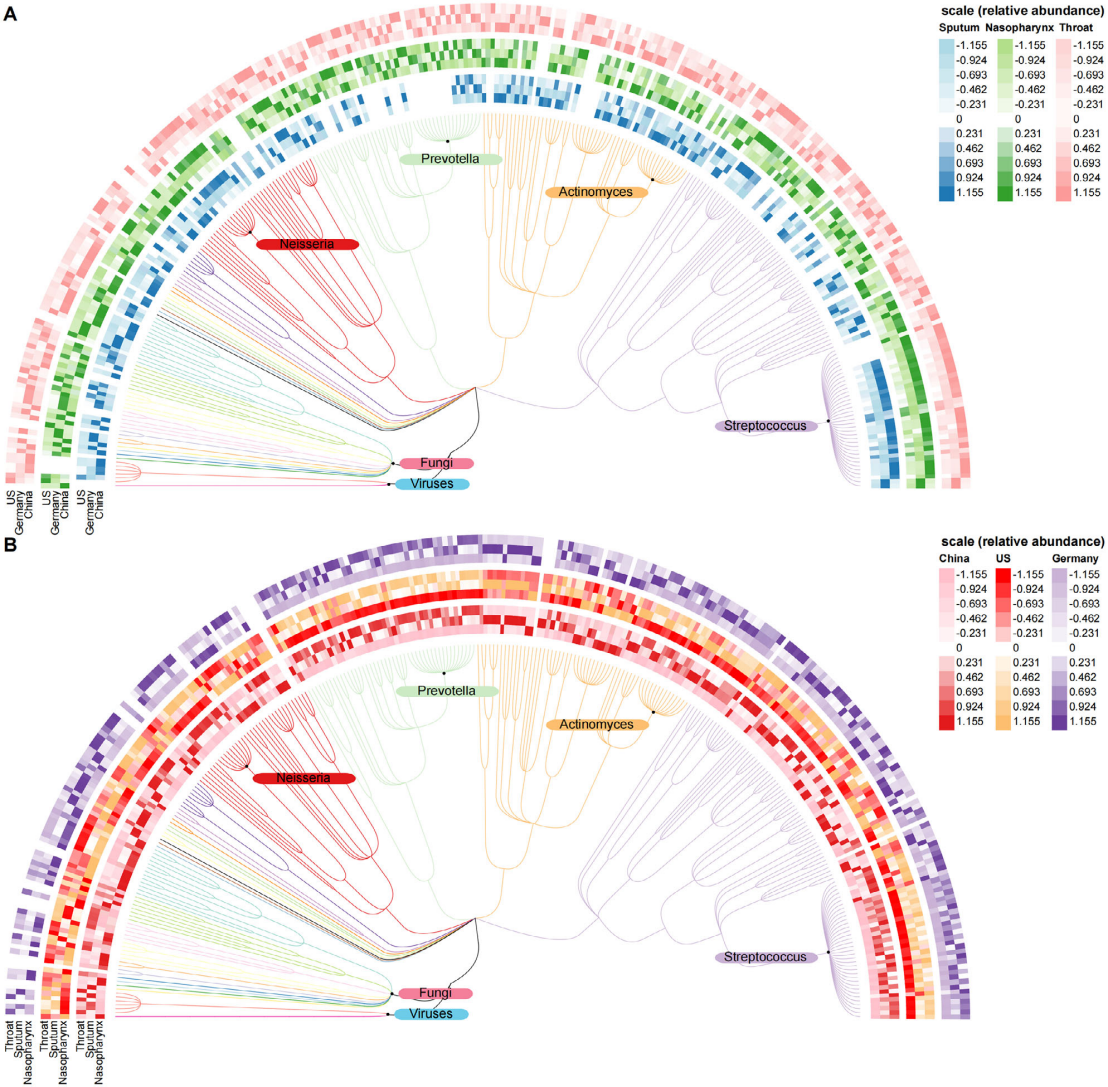
Phylogenetic tree and relative abundance heatmap of differentially abundant species across countries and sampling sites. (A) Site‐based differential species analysis. The central phylogenetic tree shows evolutionary relationships of species exhibiting significant abundance differences across countries. The outer ring heatmap represents the relative abundance (row‐standardized) of these differentially abundant species in samples from each country. Blue: Sputum; green: Nasopharynx; pink: Throat (country‐specific differences within each site). (B) Country‐based differential species analysis. The central phylogenetic tree shows evolutionary relationships of species exhibiting significant abundance differences across sampling sites. The outer ring heatmap represents the relative abundance (row‐standardized) of these differentially abundant species in samples from each site. Red: China; orange: US; purple: Germany (site‐specific differences within each country).

### 3.4 Antibiotic Resistance Genes Across Sampling Sites and Geographic Regions

Given the clinical relevance of antibiotic resistance, we specifically investigated ARGs in human airway metagenomes. Using the CARD database, we identified a total of 1,095,568 potential ARGs, accounting for 4.9% of the total gene abundance. These ARGs corresponded to 2,412 antibiotic resistance ontology (ARO) terms, covering resistance against antibacterial agents across 44 distinct drug classes. The most abundant resistance types were those against tetracyclines (0.93%), macrolide (0.93%) and fluoroquinolone (0.9%) (Figure 4A). We next investigated the taxonomic origins of these major resistance determinants. As shown in Figure 4B, ARGs conferring resistance to macrolides, tetracyclines, and fluoroquinolones were predominantly encoded by *Streptococcus*, with *Actinomyces* and *Veillonella* as secondary contributors. This underscores the central role of *Streptococcus* in shaping the antibiotic resistance potential of the airway microbiome.

**FIGURE 4:**
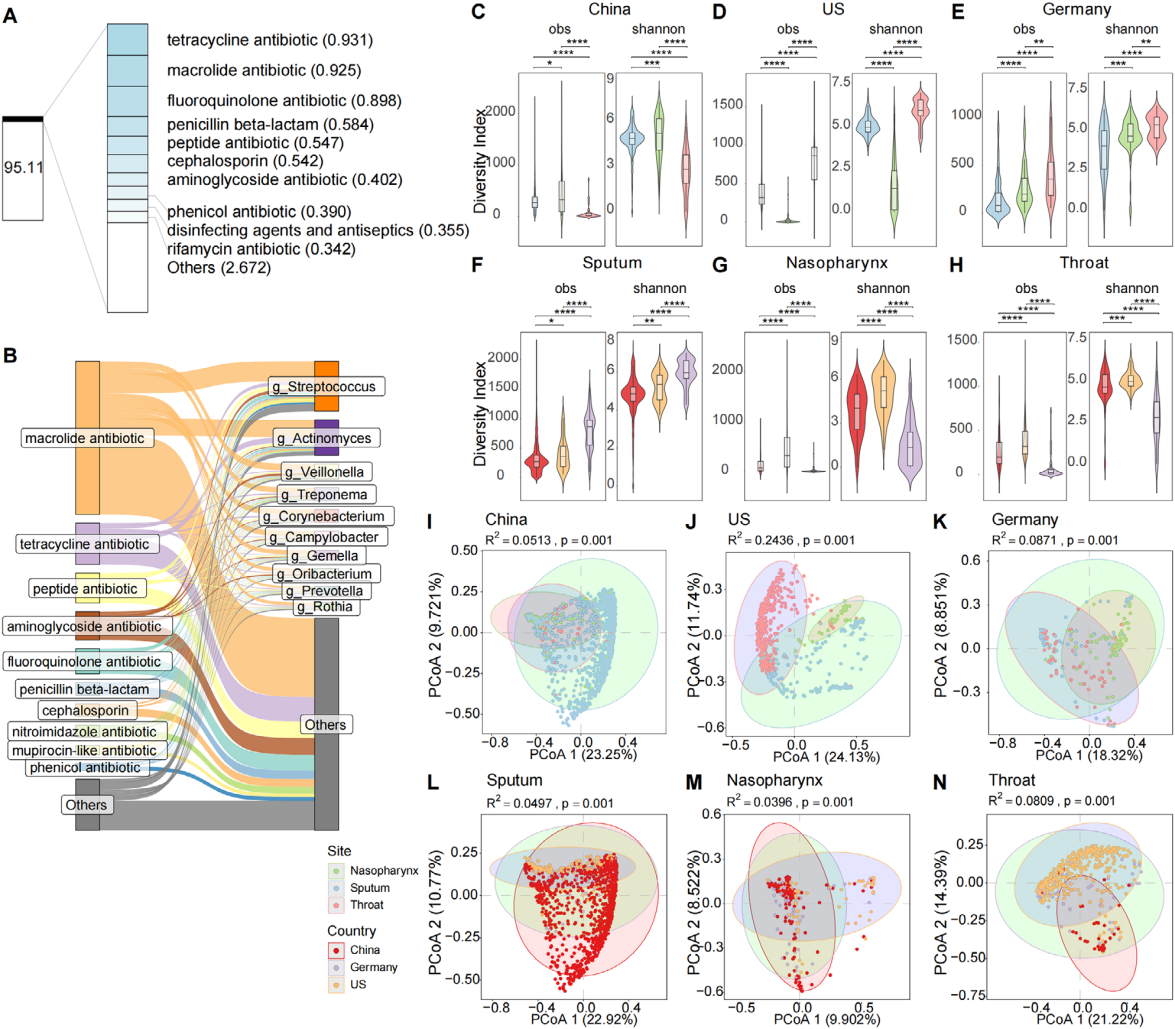
Antibiotic resistance gene (ARG) features and diversity analysis. (A) Distribution of ARGs by antibiotic class. (B) Sankey diagram showing the association network between major antibiotic classes and bacterial genera. (C–E) Comparisons of ARG alpha diversity (Observed features and Shannon index) across different sampling sites within each country. (F–H) Comparisons of ARG alpha diversity across different countries within each sampling site. Asterisks indicate statistically significant differences (*p < 0.05, **p < 0.01, ***p < 0.001, ****p < 0.0001). (I–K) PCoA plots based on Bray‐Curtis distances for different sampling sites within each country. (L–N) PCoA plots based on Bray‐Curtis distances for different countries within each sampling site.

We then assessed the overall influence of disease status, geographic origin, and anatomical site on the resistome structure. PERMANOVA of ARG profiles showed that disease status significantly affected the resistome across most sites, especially in oropharynx and cough swabs (Figure S3A). This effect was geographically heterogeneous, being strongest in Switzerland and the UK but not significant in Italy (Figure S3B). Alpha diversity analysis further revealed heterogeneity in ARG richness and evenness (Figure 4C‐H). China consistently exhibited the lowest Shannon diversity (*p* < 0.0001), whereas the US showed higher richness and Shannon diversity in both throat and nasopharyngeal samples. Across the US and Germany, throat samples consistently harbored the highest richness and diversity, followed by nasopharynx and sputum, suggesting that the upper airway serves as a primary resistance reservoir due to environmental exposure and oral microbiota interactions. PCoA confirmed significant stratification by both geography and site (Figure 4I‐N). Samples from China, the US, and Germany formed distinct clusters, with the strongest separation in the US cohort (R^2^ = 24.4%, *p* = 0.001), followed by Germany (R^2^ = 8.7%) and China (R^2^ = 5.1%). Similarly, sputum, nasopharynx, and throat separated into discrete clusters across all countries.

We next examined the distribution of shared and unique ARGs across sites and countries (Figure S3C‐H). Across all three countries, most ARGs were shared among throat, nasopharynx, and sputum, with China sharing 1,210 AROs, the US sharing 964 AROs, and Germany sharing 1,148 AROs. When stratified by anatomical site, sputum samples displayed the highest cross country conservation (1,364 AROs shared among all three nations), indicating a conserved lower airway resistome. In contrast, the nasopharynx showed greater geographic variability (950 shared terms; 436 unique to China versus 28 to Germany), and the throat exhibited marked divergence (1,138 shared; 210 unique to the US versus 22 to 37 in other countries). These findings underscore that the lower airway resistome is highly conserved across countries, whereas the upper airway reflects more pronounced geographic heterogeneity in ARG composition.

Finally, the heatmap illustrated the detailed distribution of ARG categories across anatomical sites and geographic regions (Figure S3I). Resistance to tetracyclines, macrolides, fluoroquinolones, and penicillins or beta lactams was ubiquitous and highly abundant across all samples, whereas aminoglycosides and carbapenems showed relatively lower prevalence. Anatomically, throat samples generally exhibited a higher ARG burden compared to nasopharyngeal and sputum samples, likely reflecting the complex oral reservoir. Geographically, Chinese cohorts displayed markedly elevated levels of resistance to tetracyclines, macrolides, and fluoroquinolones relative to the US and German samples, suggesting regional differences in antibiotic usage or environmental pressure. These findings highlight that although core resistance mechanisms are conserved across the respiratory tract, the resistome composition is significantly modulated by local ecological and geographical factors.

### 3.5 Virulence Factor Genes Across Sampling Sites and Geographic Regions

Given the clinical relevance of virulence factors in airway pathogenesis, we profiled virulence‐associated genes in human airway metagenomes. We characterized 9,014 virulence‐associated genes, corresponding to 45,263 unique VFGs, which were classified into 13 functional categories. The most abundant categories were immune modulation (0.07%), followed by nutritional/metabolic factor (0.05%) and adherence (0.04%) (Figure 5A). Taxonomic attribution (Figure 5B) revealed that immune modulation and adherence were the dominant virulence strategies, primarily attributed to *Haemophilus* and *Streptococcus*.

**FIGURE 5:**
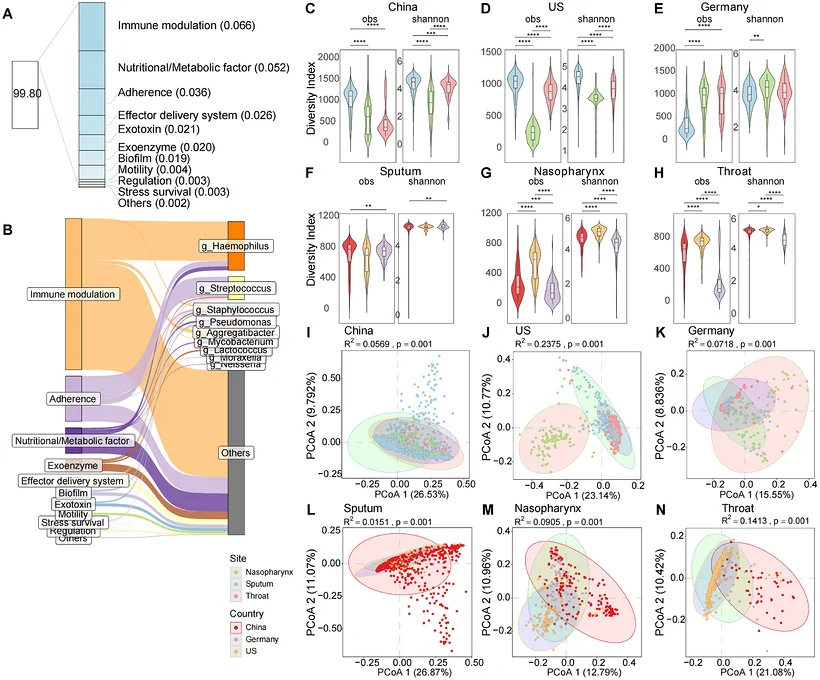
Virulence factor gene (VFG) features and diversity analysis. (A) Distribution of VFGs by functional category. (B) Sankey diagram showing the association network between major virulence factor categories and bacterial genera. (C–E) Comparisons of VFG alpha diversity across different sampling sites within each country. (F–H) Comparisons of VFG alpha diversity across different countries within each sampling site. Asterisks indicate statistical significance levels (*p < 0.05, **p < 0.01, ***p < 0.001, ****p < 0.0001). (I–K) PCoA plots based on Bray‐Curtis distances for different sampling sites within each country. (L–N) PCoA plots based on Bray‐Curtis distances for different countries within each sampling site.

We then assessed the influence of disease status, geographic origin, and anatomical site on the virulome structure. PERMANOVA of VFG abundance showed that disease status significantly affected functional profiles, with the strongest explanatory power in cough swabs, oropharynx, and sputum samples (Figure S4A). This effect was geographically heterogeneous, with high variance explained in Switzerland and Germany but non‐significant associations in Italy, the UK, and the US (Figure S4B), suggesting that host‐pathogen interactions driving virulence are modulated by local environmental or genetic factors.

Alpha diversity analysis (Figure 5C‐H) revealed that the US cohort exhibited the highest VFG richness and evenness across nasopharynx and throat samples (*p* < 0.0001). Within China and the US, sputum samples consistently harbored a richer VFG repertoire than upper airway sites (nasopharynx and throat), suggesting that the lower airway supports a more complex array of pathogenicity mechanisms. PCoA further demonstrated distinct clustering of VFG profiles by both country and anatomical site (Figure 5I‐N). Notably, anatomical site explained a larger proportion of variance in virulence gene profiles compared with some geographic comparisons, underscoring strong local microenvironmental selection on virulence potential.

We next examined the distribution of shared and unique VFGs across sites and countries (Figure S4C‐H). Across all countries, a large proportion of VFGs were ubiquitous within specific anatomical niches. In China, 5,113 VFGs were shared between nasopharynx and sputum, with sputum harboring the highest number of unique VFGs (2,640 VFGs), suggesting enrichment of specific virulence mechanisms in the lower airway. In comparison, the shared core virulomes of the US and Germany were smaller (810 and 1,630 VFGs, respectively). When stratified by anatomical site, sputum samples displayed the highest cross‐country conservation (2,237 shared VFGs), indicating a globally consistent lower airway virulome. In contrast, the nasopharynx and throat showed greater geographic variability (e.g., throat had 2,762 unique VFGs in the US versus 469 to 209 in the China and Germany).

The heatmap of virulence factor functional categories (Figure S4I) revealed distinct patterns driven by both anatomical niche and geographic origin. Adherence factors consistently exhibited high relative abundance across all cohorts, underscoring their fundamental role in colonization. Geographically, German samples generally maintained higher levels of immune modulation and exoenzyme activity, whereas the US nasopharynx samples displayed exceptionally high enrichment of nutritional/metabolic factors (27.1%) compared with other groups. In the specific context of BALF (Figure S4J), the functional profile shifted toward mechanisms of host interaction; notably, the US samples were characterized by significantly higher abundances of exotoxins (8.0%) compared with Chinese samples, which instead prioritized nutritional/metabolic factors and immue modulation. Collectively, these results suggest that although core colonization functions like adherence are conserved, specific virulence strategies, ranging from metabolic adaptation in the upper airways to toxin mediated interactions in the lungs, are dynamically shaped by regional environmental pressures and local microenvironments.

### 3.6 Antimicrobial Peptides Across Sampling Sites and Geographic Regions

After elucidating the antibiotic resistance and virulence potential of the airway microbiome, we further explored the distribution of antimicrobial peptides (AMPs) with potential therapeutic value within this gene set. A total of 46,134 AMP‐encoding sequences were identified, the majority of which could not be assigned to known species (Figure 6A). Only 566 sequences were assigned to common respiratory microbiota genera such as *Streptococcus*, *Prevotella*, and *Clostridium*.

**FIGURE 6:**
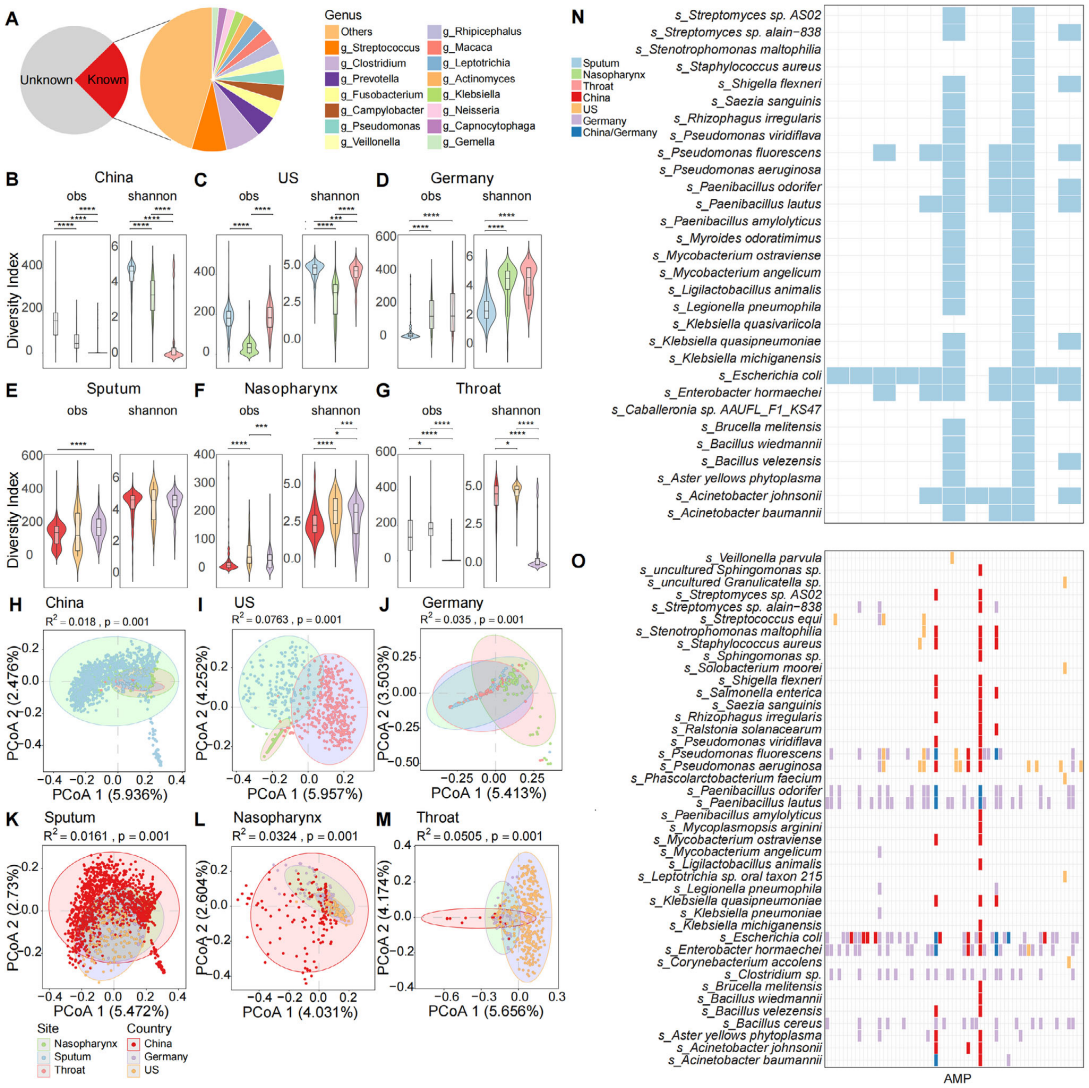
Antimicrobial peptide (AMP) features, diversity, and species association analysis. (A) Genus‐level composition of AMP origins. Left pie chart shows the proportion of known versus unknown AMP origins. The right donut chart further dissects the relative abundances of bacterial genera among known sources. (B–D) AMP gene diversity across different sampling sites within each country. (E–G) AMP gene diversity across different countries within each sampling site. (H–J) PCoA plots based on Bray‐Curtis distances for different sampling sites within each country. (K–M) PCoA plots based on Bray‐Curtis distances for different countries within each sampling site. (N) Heatmap of species negatively correlated withAMPs insputum samples (blue). No significant correlations were detected in nasopharygeal (green) or throat (pink) samples. (O) Heatmap of species negatively correlated with AMPs across different countries (China in red, US in orange, Germany in purple).

We then assessed the influence of disease status, geographic origin, and anatomical site on the AMP repertoire. PERMANOVA based on AMP annotations revealed that disease status significantly shaped the AMP community across most sites, with the strongest explanatory power observed in oropharynx, cough swabs, and BALF samples (Figure S5A). This disease‐driven separation exhibited marked geographical heterogeneity (Figure S5B), being highly significant in Switzerland and the UK but largely undetectable in Italy, implying that the immunomodulatory landscape of the airway microbiome varies substantially across different populations.

Alpha diversity analysis (Figure 6B‐G) revealed significant variations in AMP richness and evenness. Geographically, the US samples generally showed higher observed AMP counts and Shannon diversity than German and Chinese samples, with a more even distribution of AMP types. Anatomically, sputum samples consistently harbored a significantly richer AMP repertoire than upper airway sites (nasopharynx and throat) across China and the US, indicating that the lower airway selects for a broader array of antimicrobial defenses. PCoA based on Bray‐Curtis dissimilarity (Figure 6H‐M) further demonstrated clear structural separation of AMP profiles by both geography and anatomical site. Within each site, samples clustered distinctly by country, with the strongest separation in throat (non‐overlapping clusters among China, the US, and Germany). Within each country, samples separated clearly by anatomical site, confirming that niche environment is a primary driver of AMP variation.

We next examined the distribution of shared and unique AMPs across sites and countries (Figure S5C‐H). Within each country, AMP diversity was largely shared across the respiratory tract rather than site‐specific. For example, in China, 1,210 AMPs were shared among all three sites, while only 329 AMPs were unique to sputum. Similar patterns were observed in the US (shared: 964 AMPs) and Germany (shared: 1148 AMPs). In contrast, when stratified by site, cross‐national conservation was comparatively lower, suggesting that geography may contribute more to AMP heterogeneity than anatomical location.

Correlation analysis between AMP abundance and bacterial taxa (Figure 6N,O) identified 326 significant negative associations (Spearman's |*ρ*| > 0.4) involving 2,617 AMPs and 4,055 bacterial species, suggesting a potential inhibitory role of AMPs in shaping the microbiome. These interactions exhibited distinct site‐specific and geographical patterns. Anatomically, many negative correlations were enriched in sputum samples; for instance, *Escherichia coli* showed extensive negative associations with multiple AMPs in this niche. Geographically, the interaction networks varied by country: in China, *E. coli* was negatively correlated with a broad spectrum of AMPs; in the US, *Pseudomonas aeruginosa* emerged as a key taxon interacting with multiple AMPs; while in Germany, significant negative associations were observed with *Paenibacillus odorifer*, *Paenibacillus lautus*, *E. coli*, *Enterobacter hormaechei*, *Clostridium* *sp*., and *Bacillus cereus*.

## 4 Discussion

The human airway microbiome plays a critical role in respiratory health and could influence the development and progression of various respiratory diseases [32]. The iHAMGC provides a comprehensive resource for studying the airway microbiome, encompassing strains with diverse population frequencies and abundances, functional elements such as ARGs, VFGs, and AMPs. This information is essential for developing effective treatment strategies and managing antibiotic resistance.

Using the iHAMGC, we mapped the taxonomic composition of key microbial taxa across sampling sites and geographic regions, revealing clear gradients from the nasopharynx to the lower airways (Figure 2; Figure S2). These gradients reflect both niche adaptation and regional variation. For bacterial communities, the nasopharynx was enriched in potential opportunistic taxa, including *Dolosigranulum pigrum* and *Clostridium* *sp*. [33, 34], alongside specific viral families. In contrast, the throat harbored diverse oral commensals and anaerobes, such as *Prevotella histicola* and *Actinomyces graevenitzii* [35]. Sputum samples, representing the lower airways, displayed a mixed signature of taxa derived from the oral cavity and mucosa‐adherent species, along with the highest fungal richness and antimicrobial peptide diversity. These spatial patterns are consistent with established physiological gradients along the respiratory tract, where temperature, pH, oxygen partial pressure, and relative humidity collectively shape niche specific growth conditions [36]. For fungal communities, the respiratory microbiome is not a random assemblage but a structured ecosystem shaped by both host microenvironments and geographic factors. *Mucoraceae* was ubiquitous across all sites, suggesting its role as a core fungal component of the respiratory tract (Figure S2). However, its abundance varied markedly, peaking in the throat while yielding to *Malasseziaceae* in the nasopharynx, indicating distinct niche partitioning. The high prevalence of skin‐associated *Malasseziaceae* in the nasopharynx likely reflects anatomical continuity with the nasolabial folds [37], whereas the dominance of *Mucoraceae* in the throat may be driven by specific aerodynamic or nutrient conditions. Geographically, the fungal community showed notable stratification, with a balanced signature in the German cohort and *Mucoraceae* dominance in the US (Figure S2), suggesting that environmental exposures such as climate, air quality, or diet modulate these local communities. For the viral community, we observed a marked shift from diverse eukaryotic viruses (e.g., *Orthoherpesviridae*, *Polyomaviridae*, *Adenoviridae*) in the nasopharynx to bacteriophage enrichment (e.g., *Herelleviridae*, *Drexlerviridae*) in the lower airways. This pattern aligns with the understanding that bacteriophages constitute the majority of the healthy lung virome and play a crucial role in shaping bacterial communities through direct interactions with hosts, including biofilm‐forming pathogens such as *Pseudomonas aeruginosa* [38, 39]. Collectively, these site‐specific signatures across bacterial, fungal, and viral communities underscore that the respiratory tract is not a single ecological entity but rather a continuum of distinct microenvironments, each shaped by local physicochemical conditions, host anatomy, and geographic influences.

Functional annotation of the airway metagenome revealed that metabolism, particularly purine, pyrimidine, and porphyrin pathways, dominates the functional landscape, indicating active nucleotide synthesis and energy production essential for microbial survival in this dynamic niche (Figure 1H). Despite vast differences in genome size, bacteria and fungi share a substantial core of KOs (19.6%) involved in fundamental life processes, whereas viruses contribute few unique functions, reflecting their reliance on host machinery (Figure S1A). PCoA confirmed clear separation by both country and site, with the US samples and throat samples showing the highest explanatory power (Figure S1E). These findings underscore that the functional potential of the airway microbiome is not uniform but rather a product of complex interactions among host health, anatomical niche, and geographic background.

Building on this functional framework, we further investigated three clinically relevant functional modules, including ARGs, VFGs, and AMPs, to understand their distribution across airway sites and geographic regions. The antibiotic resistome was dominated by tetracycline, macrolide, and fluoroquinolone resistance genes, primarily encoded by *Streptococcus* (Figure 4A,B). This pattern aligns with previous findings of a conserved airway resistome enriched in macrolide, beta lactam, fluoroquinolone, and tetracycline resistance, with *Streptococcus* and *Actinomyces* identified as key reservoirs of macrolide resistance [40]. Anatomically, sputum showed higher cross country conservation of ARGs, suggesting that the lower airway resistome is more stable and universally present. Geographically, the high ARG richness in China, combined with elevated multidrug resistance, may reflect country specific antibiotic consumption patterns, which are known to be high for broad‐spectrum agents in some regions. Virulence factor profiles were dominated by immune modulation and adherence functions, with *Haemophilus* and *Streptococcus* as major carriers (Figure 5A,B). This is consistent with the recognized roles of *Streptococcus pneumoniae* and *Haemophilus influenzae* in employing diverse virulence mechanisms to establish respiratory infections [41]. Anatomically, sputum samples harbored the richest and most unique VFG repertoires in China, suggesting that the lower airway microenvironment selects for a more complex pathogenic potential. The strong anatomical stratification implies that virulence gene distribution is not random but finely tuned to the local host‐microbe interface. The enrichment of stress survival, exotoxin, and biofilm genes in Chinese sputum samples points to a more aggressive or metabolically active community in this population, warranting further clinical investigation.

In recent years, the growing threat of antibiotic resistance has positioned AMPs a focal point in anti‐infective research. Advances in structural biology and artificial intelligence have greatly accelerated the discovery and design of synthetic AMPs, providing abundant candidate for clinical development [42, 43]. Concurrently, the mining of naturally occurring AMPs from the human microbiome has gained considerable attention. For instance, Ma and colleagues identified 2,349 candidate AMPs from the human gut microbiota and experimentally validated the antimicrobial activity of 181 peptides, highlighting their therapeutic potential [44]. AMPs are traditionally viewed as host‐derived innate immune effectors. However, our metagenomic analysis also identified a substantial microbial‐encoded AMP repertoire in the airway, comprising 46,134 sequences (Figure 6). The majority of these remained unassigned to known taxa, underscoring the vast “dark matter” of the respiratory metagenome. Anatomically, sputum harbored the richest and most cross‐nationally conserved AMP set, consistent with the lower airway's need for broad antimicrobial protection. Correlation analysis revealed 326 significant negative associations between AMPs and bacterial taxa, many of which were site‐ and country‐specific. This aligns with the established role of AMPs as critical host‐derived regulators of mucosal microbiota and modulators of inflammation [37]. Notably, the enrichment of negative correlations in sputum suggests that the lung environment imposes stronger selective pressure via AMPs than the upper airway.

Several limitations should be acknowledged. First, although we integrated a large‐scale airway metagenomic dataset, the sample distribution remained uneven across countries, sampling sites, and disease contexts, and metadata completeness varied among public cohorts. We therefore used stratified comparisons and covariate‐adjusted models where possible, but residual confounding by cohort background, sequencing protocol, host condition, medication use, and other unmeasured variables cannot be fully excluded. Second, clinical information such as antibiotic exposure, disease stage, and treatment history was not consistently available, limiting direct interpretation of ARG, VFG, and AMP profiles in relation to clinical phenotypes. Third, respiratory samples, especially low‐biomass samples, may be affected by environmental, reagent, or food‐associated contamination; therefore, low‐abundance signals, including genes annotated to *Plasmodium ovale*, should be interpreted cautiously. Finally, taxonomic and functional annotations depend on reference databases and computational prediction, and predicted ARGs, VFGs, and AMPs represent genetic potential rather than experimentally validated resistance, virulence, or antimicrobial activity. These limitations should be considered when using the iHAMGC as a reference resource and when interpreting site‐ or geography‐associated microbial features.

## 5 Conclusions

In summary, we have established the iHAMGC, a comprehensive metagenomic gene catalog that significantly expands the known taxonomic diversity and functional profiles of the human airway microbiome. Leveraging this resource, we systematically characterized the distribution of key microbial biomarkers across airway compartments and geographical regions, as well as the functional landscape of ARGs, VFGs, and AMPs. The iHAMGC thus provides a foundational reference framework for future studies investigating the functional attributes and ecological dynamics of the airway microbiome.

## Author Contributions

X.Z. and B.C. conceived and managed the study. Q.Z., D.L., Y.Z., M.L., R.G., and Y.N. performed bioinformatic analysis and visualization. B.L., S.C., B.N., and L.Q. performed sample collection and experiments. G.X., H.D., Q.Y., and S.L. contributed to data processing and methodology. Q.Z., M.L., and X.Z. wrote the manuscript. R.G. helped to draft the manuscript. All authors contributed to data interpretation, and read and approved the final manuscript.

## Conflicts of Interest

The authors declare no conflicts of interest.

## Supporting information




**Supporting File 1**: advs76589‐sup‐0001‐SuppFigures.docx.

**Supporting File 2**: advs76589‐sup‐0002‐SuppTables.xlsx.

## Data Availability

The data that supports the findings of this study are available in the supplementary material of this article.
