# Supplementary material for: A Comprehensive Microbial Gene Catalog of the Human Airway Microbiome Across Anatomical Sites and Geographic Regions
Source: Adv Sci (Weinh). 2026 Jul 27:e76589. Online ahead of print. doi: 10.1002/advs.76589 (PMC13403716; doi:10.1002/advs.76589)
Supplement: Supplementary file 1 — Supporting File 1: advs76589‐sup‐0001‐SuppFigures.docx. [file ADVS-9999-e76589-s001.docx]

**A comprehensive microbial gene catalog of the human airway microbiome across anatomical sites and geographic regions**

Supplementary document


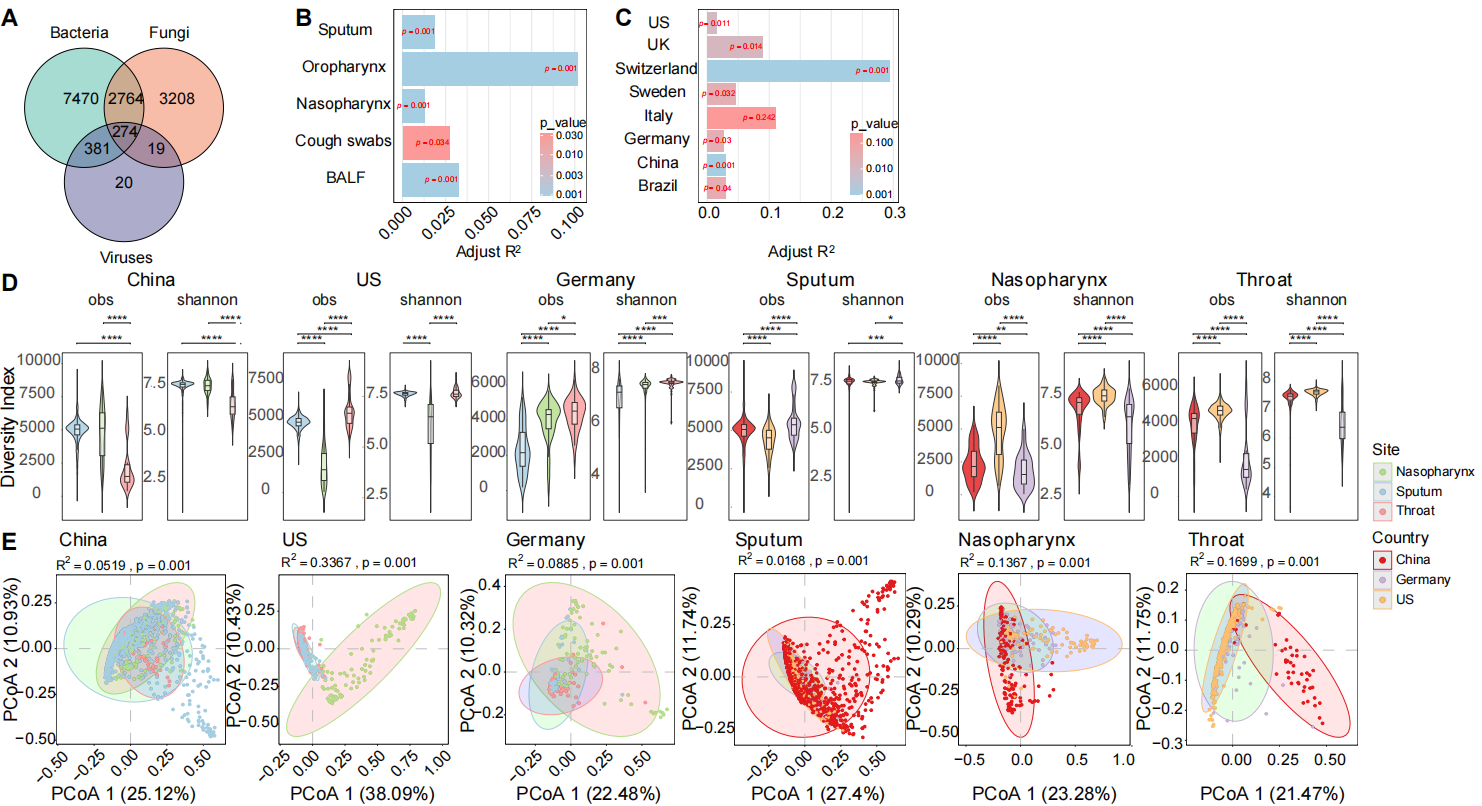


**Figure S1. Functional profiles and diversity analysis based on KEGG orthologs (KOs).**  **(A)** Venn diagram showing KO gene overlaps among prokaryotes, eukaryotes, and viruses. **(B)** Explanatory power of disease status on KEGG functions across different sampling sites (PERMANOVA). **(C)** Explanatory power of disease status on KEGG functions across different countries (PERMANOVA). **(D)** Comparisons of alpha diversity of KO functional genes across different groups. The left three subplots correspond to China, US, and Germany, showing differences in Observed features and Shannon index across sampling sites within each country; the right three subplots correspond to Sputum, Nasopharynx, and Throat, showing diversity differences across countries within each site. **(E)** PCoA analysis of KO functional profiles based on Bray‑Curtis distances. The left three subplots correspond to China, US, and Germany, showing clustering of functional profiles for different sampling sites (color‑coded) within the same country; the right three subplots correspond to Sputum, Nasopharynx, and Throat, showing clustering patterns for different countries within the same site.


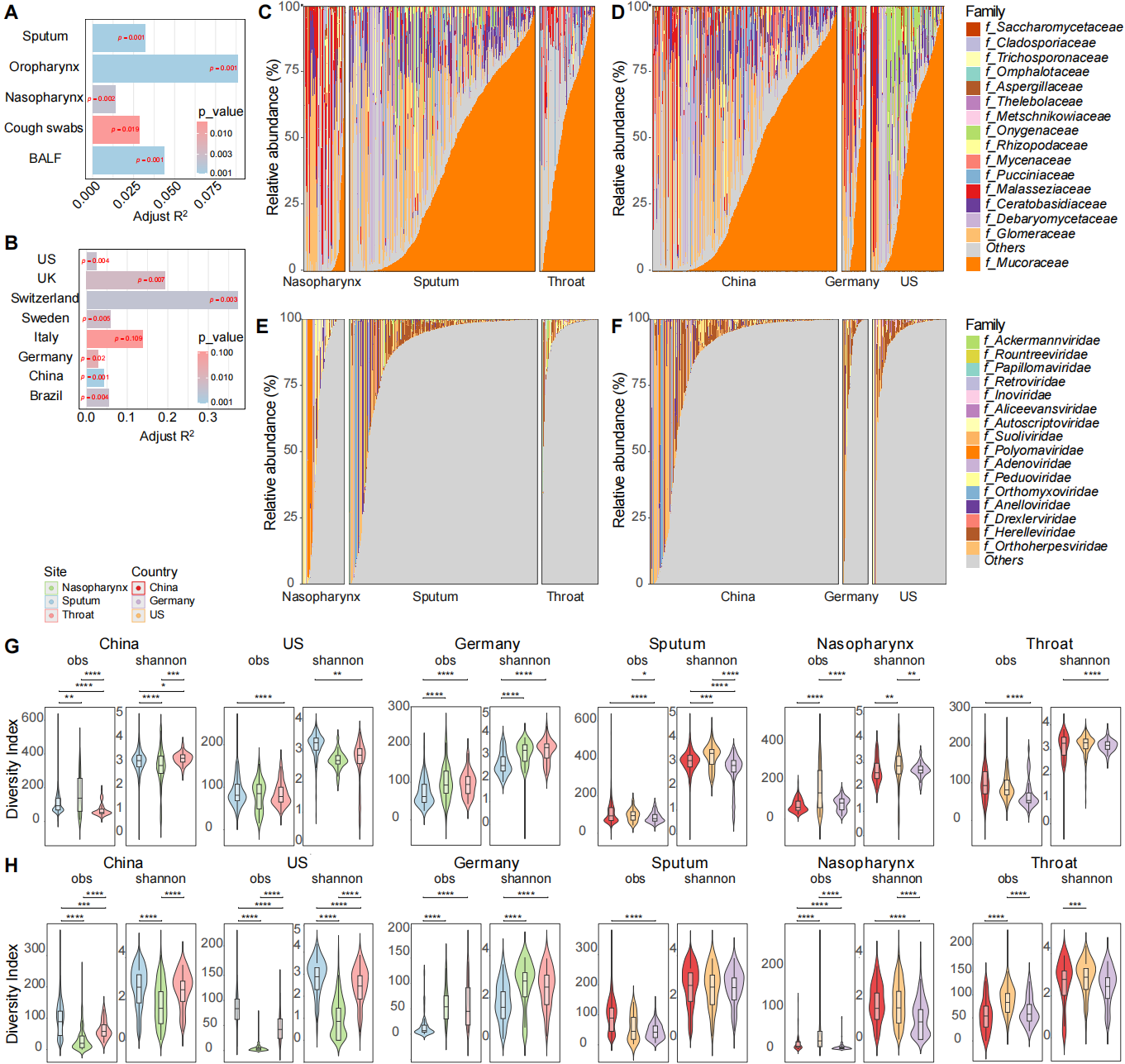


**Figure S2. Composition, structure, and diversity analysis of fungal and viral communities. (A)** Explanatory power of disease status on microbial community structure across different sampling sites (PERMANOVA). **(B)** Explanatory power of disease status on microbial community structure across different countries (PERMANOVA). **(C-D)** Stacked bar plots of fungal composition at the family level across different sampling sites (C) and countries (D). **(E-F)** Stacked bar plots of viral composition at the family level across different sampling sites (E) and countries (F). **(G)** Comparisons of alpha diversity of fungal species across different groups. The left three subplots correspond to China, US, and Germany, showing differences in Observed features and Shannon index across sampling sites within each country; the right three subplots correspond to Sputum, Nasopharynx, and Throat, showing diversity differences across countries within each site. **(H)** Comparisons of alpha diversity of viral species across different groups. The left three subplots correspond to China, US, and Germany, showing differences in Observed features and Shannon index across sampling sites within each country; the right three subplots correspond to Sputum, Nasopharynx, and Throat, showing diversity differences across countries within each site.


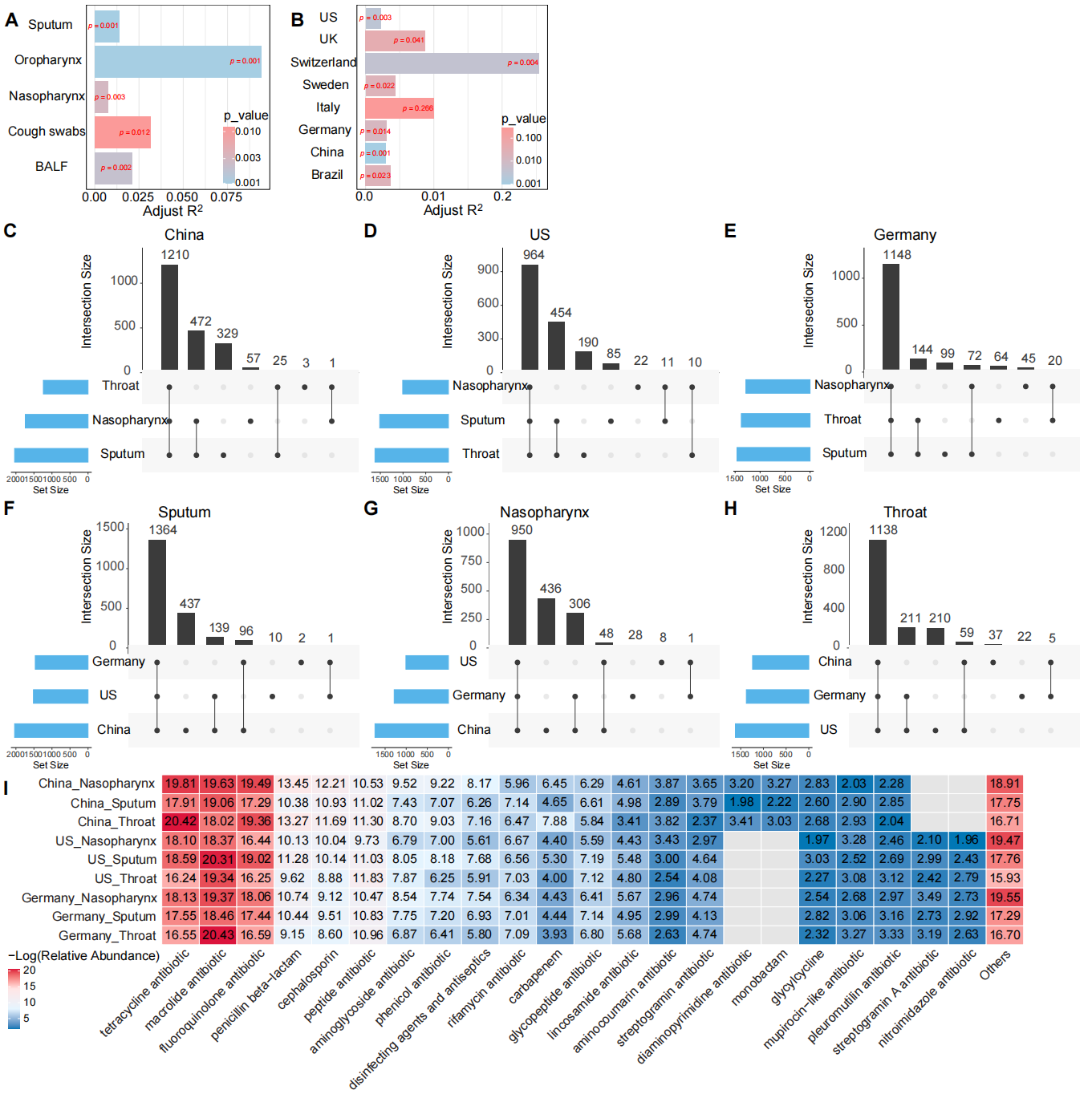


**Figure S3. Functional features and distribution patterns of antibiotic resistance genes (ARGs). (A)** Explanatory power of disease status on ARG functional profiles across different sampling sites (PERMANOVA). **(B)** Explanatory power of disease status on ARG functional profiles across different countries (PERMANOVA). **(C–E)** UpSet plots of ARGs across different sampling sites within each country. **(F–H)** UpSet plots of ARGs across different countries within each sampling site. **(I)** Heatmap of antibiotic resistance mechanism (AMR) abundances across three countries and three sites.


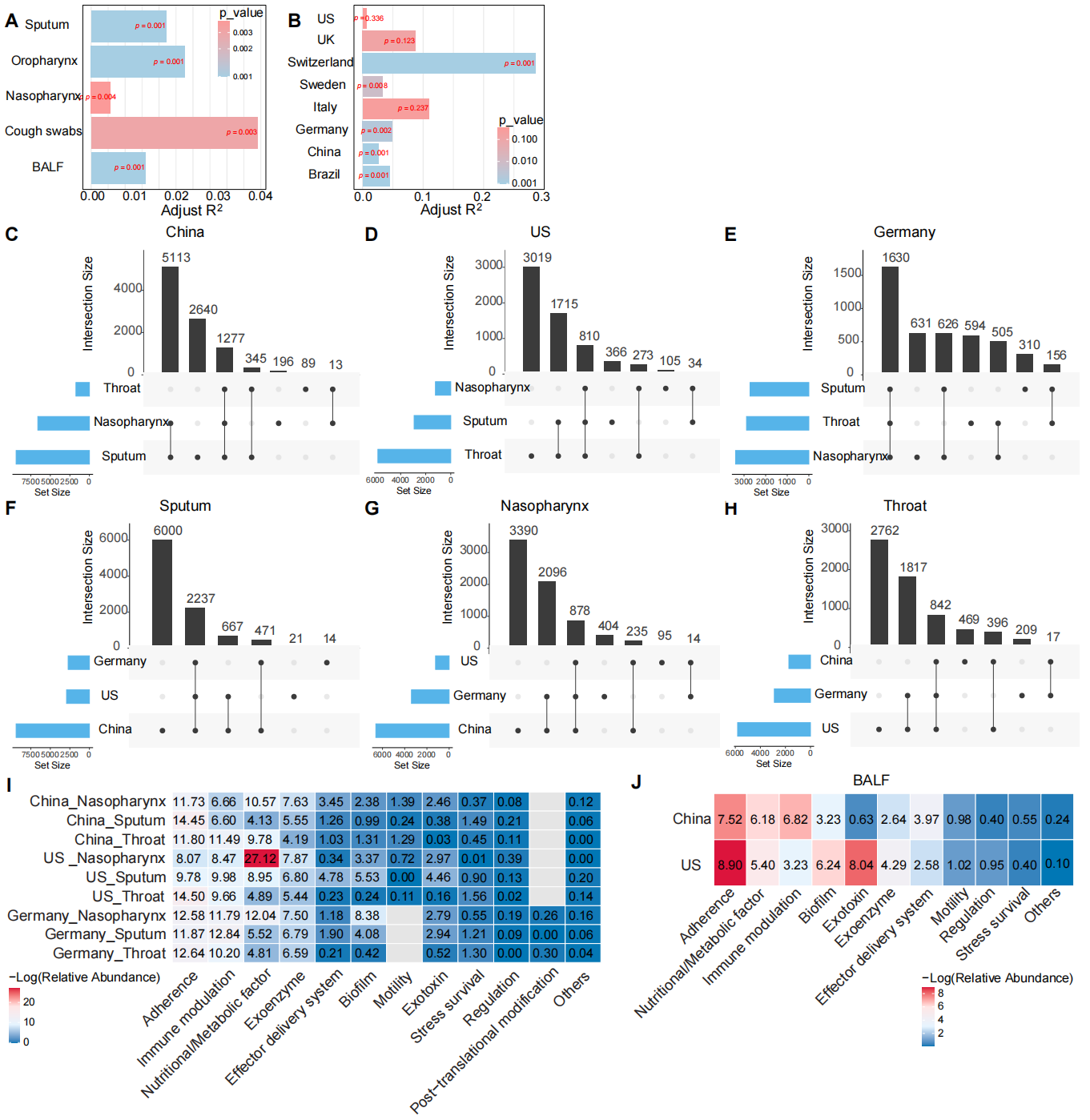


**Figure S4. Functional features, distribution patterns, and abundance analysis of virulence factor genes (VFGs). (A)** Explanatory power of disease status on VFG functional profiles across different sampling sites (PERMANOVA). **(B)** Explanatory power of disease status on VFG functional profiles across different countries (PERMANOVA). **(C–E)** UpSet plots of VFGs across different sampling sites within each country. **(F–H)** UpSet plots of VFGs across different countries within each sampling site. **(I)** Heatmap of major virulence mechanism abundances across three countries and three sites. **(J)** Heatmap of virulence mechanism abundances in bronchoalveolar lavage fluid (BALF) across different countries.


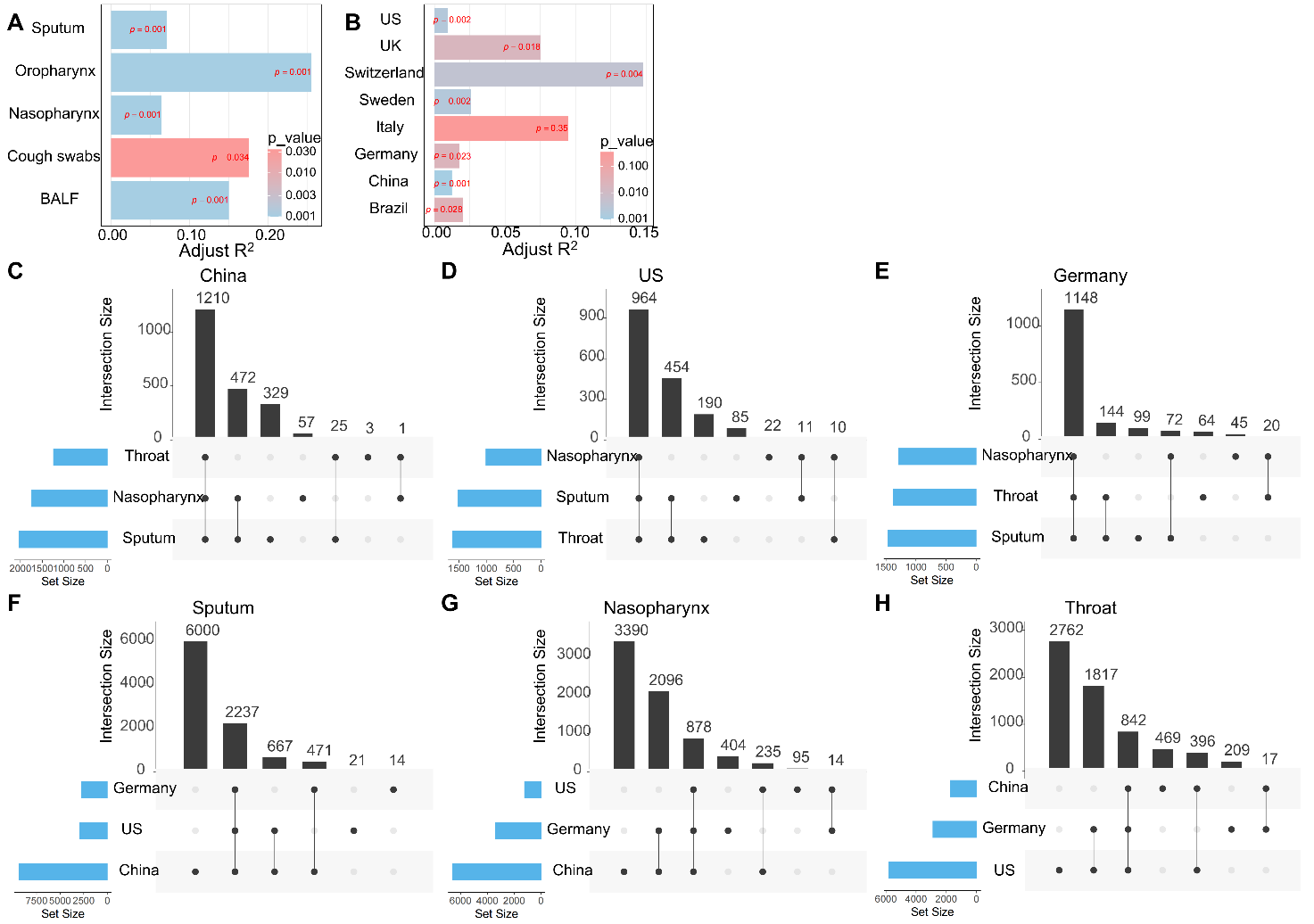


**Figure S5. Functional features, distribution patterns, and diversity analysis of antimicrobial peptides (AMPs). (A)** Explanatory power of disease status on AMP functional profiles across different sampling sites (PERMANOVA). **(B)** Explanatory power of disease status on AMP functional profiles across different countries (PERMANOVA). **(C–E)** UpSet plots of AMPs across different sampling sites within each country. **(F–H)** UpSet plots of AMPs across different countries within each sampling site.
